# Non-invasive coronary physiology based on computational analysis of intracoronary transluminal attenuation gradient

**DOI:** 10.1038/s41598-018-23134-7

**Published:** 2018-03-16

**Authors:** Yong Gyun Bae, Seung Tae Hwang, Huan Han, Sung Mok Kim, Hyung-Yoon Kim, Il Park, Joo Myung Lee, Young-June Moon, Jin-Ho Choi

**Affiliations:** 1 Computational Fluid Dynamics and Acoustics Laboratory, School of Mechanical Engineering, Korea University, Seoul, Republic of Korea; 2 Department of Radiology, Sungkyunkwan University School of Medicine, Seoul, Republic of Korea; 3 Department of Medicine, Sungkyunkwan University School of Medicine, Seoul, Republic of Korea; 4 Department of Emergency Medicine, Heart Vascular and Stroke Institute, Samsung Medical Center, Sungkyunkwan University School of Medicine, Seoul, Republic of Korea; 5 Department of Medicine, Chonnam National University Medical School, Gwangju, Republic of Korea

## Abstract

Invasive procedure is a prerequisite for studying coronary physiology. We established the measurement of non-invasive physiological parameters including coronary blood flow (CBF), flow velocity, and microvascular resistance using coronary computed tomography angiography (CCTA). Vessel-specific CBF was derived from transluminal attenuation flow encoding (TAFE) and then tested using three separate datasets consisted of computational simulation, human perfusion CT, and human CCTA. TAFE-derived CBF correlated well with measured vessel-specific myocardial blood flow and CBF. TAFE-derived CBF per myocardial mass consistently decreased with the progressive severity of stenosis, and it was found to better to detect significant stenosis than transluminal attenuation gradient (TAG). With the addition of vessel anatomy, TAFE-derived CBF could calculate flow velocity and microvascular resistance. The results of non-invasively acquired parameters according to the severity of stenosis were similar to those obtained through invasive physiology studies. Our study demonstrated that non-invasive comprehensive coronary physiology parameters can be derived from CCTA without any pre-specified condition or performing complex heavy computational processes. Our findings are expected to expand the clinical coverage of CCTA and coronary physiology.

## Introduction

The key role of coronary artery is supplying sufficient blood flow which contains vital materials such as oxygen or glucose to match the needs of subtended myocardium. Therefore, quantification of vessel-specific coronary blood flow (CBF) has paramount importance in coronary physiology. However it is limited by requirement of invasive catheterization and use of dedicated intracoronary Doppler wire or infusion catheter^1,2^.

Coronary computed tomography angiography (CCTA) is a snapshot of dynamic intraluminal contrast transit. Therefore, intracoronary hemodynamics can be calculated by applying mass conservation and enhancement dynamics used in myocardial blood flow (MBF) quantitation^3^. Briefly, absolute CBF can be calculated from the time-dependent change of contrast density proximal to coronary artery as input function of contrast cohort, arterial volume to be filled by contrast cohort, and gradient of intraluminal contrast density that reflects flow velocity.

**Figure 1:**
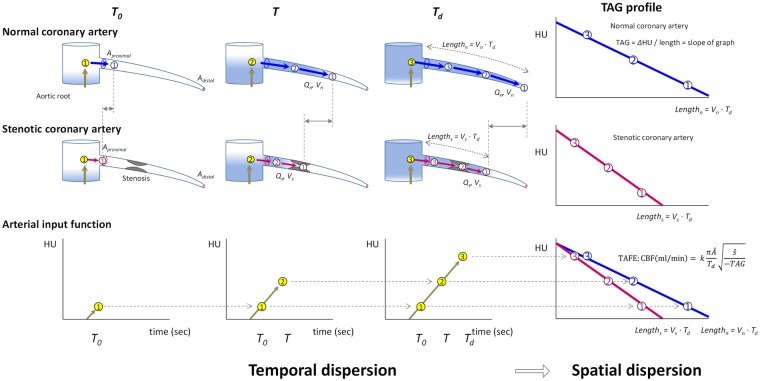
Concept of transluminal arterial flow encoding (TAFE). Contrast bolus moves slower in stenotic artery than in normal artery (points 1 to 3). Coronary CT angiography (CCTA) is a snapshot of contrast bolus advection driven by CBF at time *T*_*d*_. Vessel-specific CBF is calculated from arterial input function of contrast cohort in ascending aorta (yellow-colored numbered circles), arterial volume (the product of length and median luminal area, approximately) to be filled by the contrast cohort, and TAG (slope of intraluminal contrast density) which reflects the flow velocity. Modified from Lardo *et al*.^4^ with permission.

On the basis of this concept, Lardo *et al*.^4^ have developed an elegant hemodynamics formula named transluminal attenuation flow encoding (TAFE) (Fig. 1). In this study, we investigated and validated TAFE using human subjects data. We validated TAFE using computational flow dynamics model and human perfusion CT. Further, we examined TAFE-derived CBF, flow velocity, and microvascular resistance in CCTA according to varying severity of coronary artery stenosis to determine the feasibility of non-invasive comprehensive coronary physiology studies.

## Methods

### Computational flow dynamics model

A virtual coronary artery model with a branching pattern following Murray’s law and ipsilateral stenosis of 0–95% was established (Supplementary Figure IA,B). Intravenous contrast injection was simulated by arterial input function (AIF) which is a temporal profile of iodine mass fraction (Supplementary Figure IC–E). The Comparison of the flow ratio of stenotic vessel to normal vessel calculated by TAFE to those calculated by computational flow dynamics model validated this model (Supplementary Figure IF,G). The details are described in Supplementary Appendix.

### Patients

Between January 2011 and December 2014, we consecutively enrolled 30 perfusion CT cases without significant stenosis defined by diameter stenosis (DS) ≥ 50% and 100 CCTA cases with varying degrees of stenosis which were performed for elective evaluation of coronary artery disease. To minimize imaging bias related to temporal inhomogeneity, only CCTA acquired with single heart beat scan were enrolled. No patient was simultaneously enrolled in both studies. Patients with hypertrophic or secondary hypertrophy, prior myocardial infarction, total occlusion^5^, revascularization, heart failure, structural or congenital heart disease, prosthetic valves, or any clinical instability were not included. Institutional review board of Samsung Medical Center approved the study protocol dealing with anonymized image data; therefore, the requirement of informed consent was waived.

### CT image acquisition

Second-generation dual-source CT scanner (SOMATOM Definition Flash; Siemens Medical Solution) was used as previously described^6^. In perfusion CT, hyperemia was induced by continuous intravenous adenosine infusion of 140 μg/kg/min for 3 min. Intravenous contrast infusion included 70–80 ml of iomeprol (350 mg I/mL, Bracco) followed by 40 ml of saline at a rate of 4 to 5 ml/sec. Scan parameters were 280 msec gantry rotation, heart rate-dependent pitch 0.17–0.2, tube voltage 100 kV, and tube current 330 mA. Images were acquired for 30 sec during end inspiration. Dynamic datasets were acquired for every other R–R interval at two rapidly alternating table positions that provided 73 mm Z-axis coverage.

CCTA without hyperemic induction was performed with 2 × 128 × 0.6 mm sections and 2 × 64 × 0.6 mm detector collimation using Z-axis flying focal spot technique with prospective ECG-triggered high-pitch (pitch 3.2–3.4) helical mode. Contrast intravenous infusion consisted of 60 ml and then 40 ml of saline at a rate of 4 ml/sec. Oral beta-blocker and nitroglycerin were administered and heart rate <60/min was confirmed.

Radiation-reduction technique (REDose4D, Siemens Healthcare) was applied as reasonably as possible. The mean effective radiation dose of perfusion CT and CCTA was 5.1 and 0.9 mSv, respectively.

### CT Image reconstruction and measurement

A dedicated workstation (iNtuition, TeraRecon) was used^5–10^. In brief, CCTA and mid-diastolic images of perfusion CT were reconstructed with 0.6 mm slices. Vessel length, cross-sectional luminal area of the most proximal and distal segments, TAG defined by *Δ*intraluminal Hounsfield Unit (HU) per 10 mm vessel length, and left ventricular (LV) mass were assessed. Vessel luminal volume was approximated by applying the product of vessel length and average luminal area^4^.

### Myocardial blood flow

In perfusion CT, LV-MBF was calculated using a dedicated volumetric perfusion analysis software (Leonardo, Siemens Medical)^6^. Then vessel-specific MBF was calculated by multiplying LV-MBF and the %fractional myocardial mass (%FMM), which is a vessel-specific myocardial mass on the basis of an allometric scaling between coronary artery length and myocardial mass^7,11^. In CCTA, resting vessel-specific MBF of normal coronary artery was assumed to be 0.90 ml/g/min^12^. Right coronary artery (RCA) supplies to both right ventricle (RV) and LV. RV was assumed to possess 36% of LV mass and the same MBF per myocardial mass (g) on the basis of the results of prior magnetic resonance imaging and pre-clinical studies that have demonstrated similar MBF per myocardium (g) in both ventricles^13,14^. Therefore, CBF of RCA proximal to the posterior descending artery bifurcation (PDA) and posterolateral artery (PL) was assumed to supply both RV mass and %FMM of LV mass subtended by PDA and PL.

### Calibration and validation of TAFE formula

**Figure 2:**
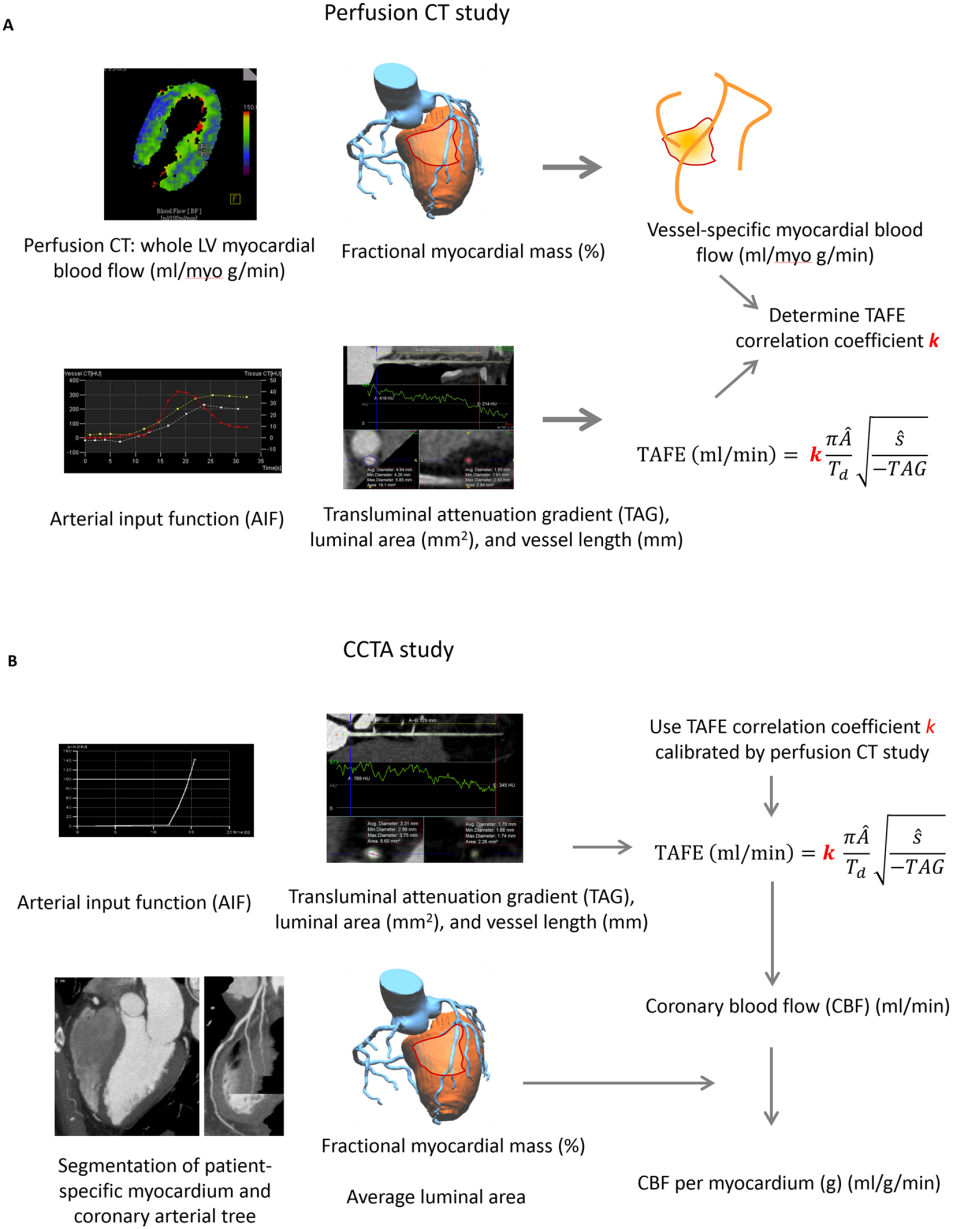
Validation and measurement of vessel-specific coronary blood flow using coronary CT angiography using TAFE formula. (**A**) In perfusion CT study, CBF was compared with vessel-specific MBF, a product of LV-MBF and %fractional myocardial mass (%FMM). TAFE coefficient ***k*** was calibrated. (**B**) CBF per myocardium (g) was calculated as the product of CBF and %FMM. Peak flow velocity was 2-fold of mean flow velocity, which is defined by dividing CBF by the average luminal area. Distal mean arterial pressure was estimated by subtracting resting whole-cycle trans-stenotic pressure gradient on the basis of the IDEAL study (1.5, 4.4, 10.8, and 29 mmHg for diameter stenosis (DS) = 0%, 1–49%, 50%–69%, and ≥70%) using mean arterial pressure, as defined by diastolic blood pressure + pulse pressure/3. Microvascular resistance calculated as the ratio of distal mean arterial pressure to the flow velocity.

With the assumption that TAG is created by the advection of contrast dye into coronary artery from AIF, TAFE calculates vessel-specific CBF (ml/min) from average cross-sectional luminal area given as Â (cm^2^), vessel length given as ŝ (cm), and time to peak enhancement given as T_d_ (min) (Equation 1; Fig. 2A)^4^.

1 $${\rm{TAFE}}:{\rm{CBF}}({\rm{ml}}/{\rm{\min }})=k\frac{\pi \hat{A}}{{T}_{d}}\sqrt{\frac{\hat{s}}{-TAG}}$$

### Flow velocity and microvascular resistance

Laminar flow and linear relationship between CBF and flow velocity were assumed on the basis of the Reynolds number [(= mean luminal diameter (m) × mean flow velocity (m/sec) × density (1060 kg/m^3^/viscosity (0.004 Pa∙sec)] <500, which is a known critical value for developing turbulent blood flow, in most vessels. Flow velocity was 2-fold of CBF divided by average luminal area^15^. Distal mean arterial pressure was estimated by subtracting assumed resting whole-cycle trans-stenotic pressure gradient on the basis of the mean arterial pressure in IDEAL study^2^. Microvascular resistance calculated as the ratio of distal mean arterial pressure to the flow velocity (Fig. 2B).

### Statistical analysis

Analyses were performed on a per-vessel basis unless specified. Categorical variables are presented as numbers and percents. Continuous variables were expressed as median with 1^st^–3^rd^ quartiles in parentheses or as mean ± SD. TAFE coefficient ***k*** was determined by linear regression estimate between CBF and vessel-specific MBF. The relationship between CBF and MBF was tested by Pearson’s correlation. Relationship among DS and physiologic parameters were interrogated with polynomial curve fitting using quadratic or cubic model, while the dose-response relation was evaluated by Jonchheere-Terpstra trend test. Two-tailed p-value < 0.05 was considered to be statistically significant. R version 3.4 (R foundation) was used.

## Results

### TAFE in computational flow dynamics model

In the virtual coronary artery model, the axial profile of iodine mass fraction consistently decreased according to the severity of stenosis. A good agreement was noted between the calculation of the flow ratio of stenotic vessel to normal vessel performed by TAFE and that by computational flow dynamics (Supplementary Appendix, Supplementary Figure IA–G).

### Patients

Both perfusion CT (n = 30) and CCTA (n = 99 after the exclusion of 1 case due to inadequate image quality) cases showed similar clinical characteristics (Table 1).

**Table 1:** Clinical characteristics of perfusion CT and CCTA cases.

	Perfusion CT (N = 30)	CCTA (N = 99)	p-value
Age (years)	66 (60–73)	62 (50–70)	0.06
Male gender	19 (63)	75 (76)	0.16
Diabetes	8 (27)	20 (20)	0.53
Hypertension	18 (60)	41 (41)	0.07
Chronic kidney disease	0 (0)	1 (1)	1.00
Current smoker	5 (17)	22 (22)	0.61
Family history of coronary artery disease	1 (3)	8 (8)	0.68
Stable angina or atypical chest pain	30 (100)	96 (97)	1.00
Unstable angina	0 (0)	3 (3)	
Significant lesions			
LAD	0 (0)	32 (32)	—
LCX	0 (0)	20 (20)	—
RCA	0 (0)	20 (20)	—

### Vessel-specific CBF, flow velocity, and microvascular resistance from TAFE

**Figure 3:**
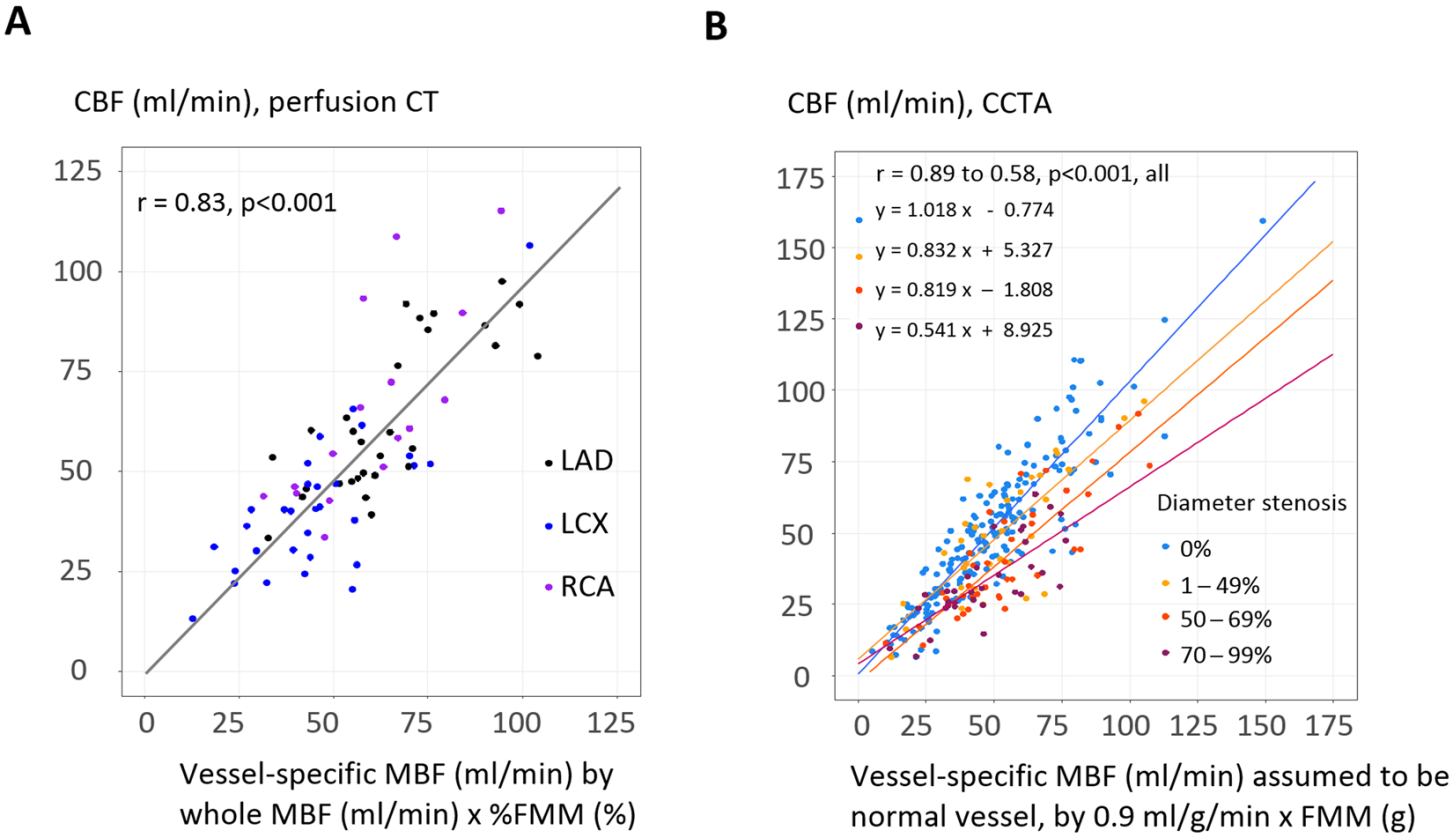
TAFE-derived coronary blood flow compared with vessel-specific myocardial blood flow. (**A**) In perfusion CT, CBF matched well with vessel-specific MBF. LAD, LCX, and RCA subgroups also showed consistent results. (**B**) In CCTA, CBF correlated well with vessel-specific MBF of assumed normal vessel calculated by MBF = 0.9 ml/g/min and FMM (g). Slopes decline with the progressive severity of stenosis.

Perfusion CT study revealed a good correlation between CBF and vessel-specific MBF derived from %FMM of the whole MBF (76 vessels, r = 0.83). All subgroup of left anterior descending, left circumflex, and RCA also showed consistent correlations between CBF and MBF (r = 0.86–0.74, all; Fig. 3A, Table 2). TAFE coefficient ***k*** was 2.793.

**Table 2:** TAFE-derived coronary blood flow in perfusion CT and CCTA.

Perfusion CT	N of vessel	CBF (ml/min)	Vessel-specific MBF, by whole MBF (ml/min) x %fractional myocardial mass	Correlation coefficient	P for correlation
All	76	51.2 (40.5–65.7)	55.3 (42.9–69.2)	0.83	<0.001
LAD	30	58.7 (48.5–84.5)	61.6 (54.8–74.4)	0.86	<0.001
LCX	30	40.3 (29.0–50.4)	43.5 (33.5–55.0)	0.76	<0.001
RCA^*^	16	59.6 (45.8–76.7)	60.5 (48.6–67.7)	0.74	<0.001
CCTA	N of vessel	CBF (ml/min)	Vessel-specific MBF assumed to be normal vessel, by 0.9 (ml/g/min) × fractional myocardial mass (g)	Correlation coefficient	P for correlation
All	290	43.7 (28.0–61.2)	47.1 (33.9–49.3)	0.84	<0.001
LAD**	98	49.6 (36.2–61.7)	54.6 (42.0–70.2)	0.71	<0.001
LCX	99	27.0 (19.1–36.5)	29.2 (21.6–41.7)	0.80	<0.001
RCA**	93	57.0 (42.7–73.6)	55.1 (46.2–66.9)	0.83	<0.001

^*^Not all RCA was available (n = 14) because the Z-axis coverage of perfusion imaging was 73 mm.

**Hypoplastic vessels (n = 7) were not included.

**Figure 4:**
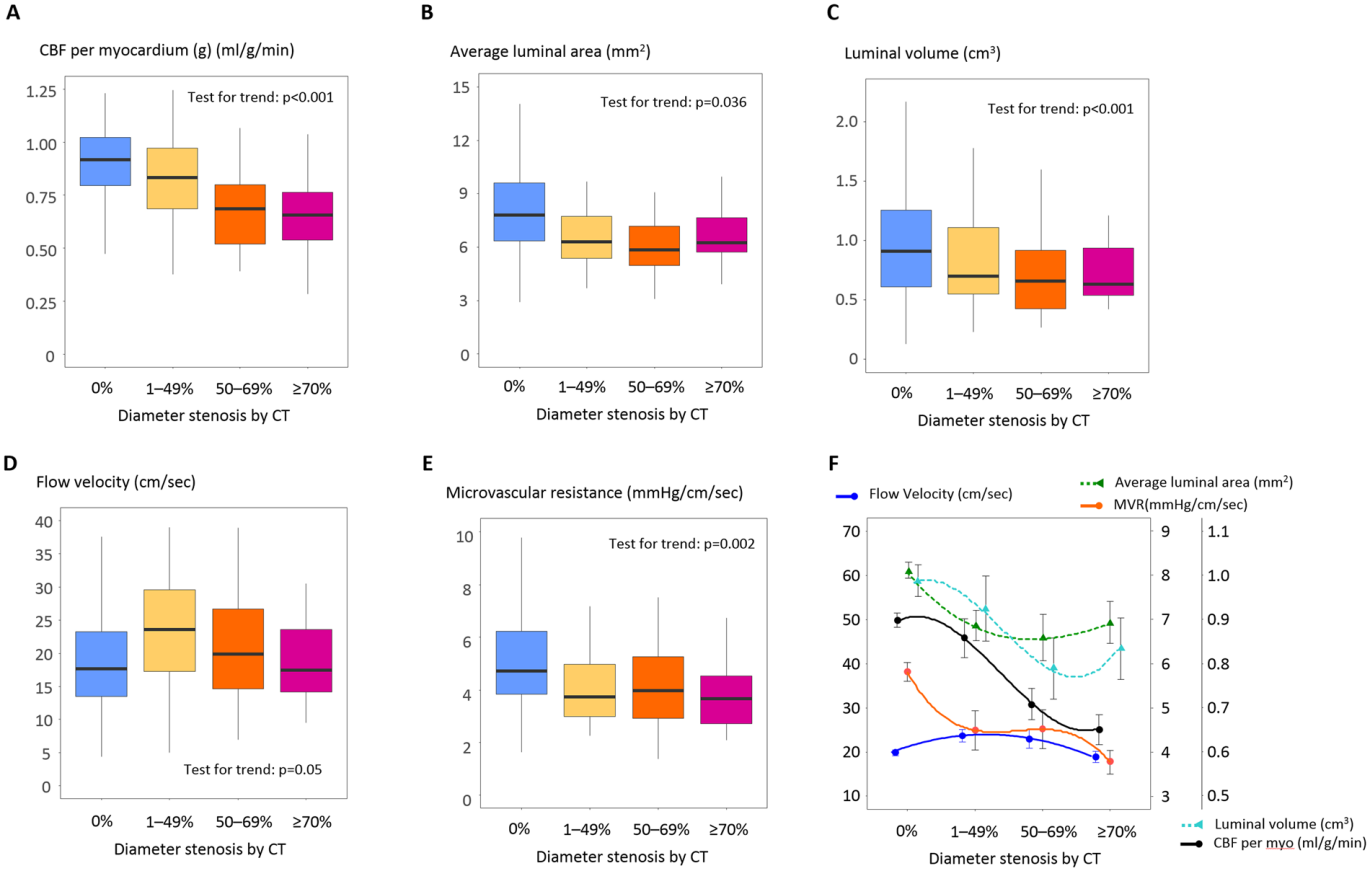
TAFE-derived coronary blood flow, flow velocity, and microvascular resistance according to diameter stenosis. CBF per myocardium (g) decreased according to the severity of stenosis (**A**–**C**). However, flow velocity did not decrease (**D**), and was accompanied by concomitant reduction of mean luminal area, volume, and microvascular resistance (**B**,**C**,**E**). Boxplots show median and 1^st^–3^rd^ quartile as box, and 1.5-fold of the interquartile range as whisker. Panel F integrates panels A–E with data as mean ± SE. Curves were fitted by polynomial quadratic models.

With this TAFE coefficient ***k***, CCTA study could successfully calculate TAFE-derived CBF in 290 vessels (Table 2). Interestingly, TAFE-derived CBF matched well with vessels of DS = 0% (slope coefficient = 1.02, r = 0.89, blue line in Fig. 3B); however, consistently decreased according to the stenosis severity (slope coefficient = 0.83–0.54; yellow, orange, and red lines in Fig. 3B). TAFE-derived CBF normalized by the subtended myocardial mass (g) consistently decreased according to the DS severity of 0% −≥70% (0.91–0.64 ml/g/min, p < 0.001; Fig. 4A). In addition, both mean luminal area and volume decreased according to the DS severity of 0% −≥70% (7.80–6.25 mm^2^; 0.94–0.74 cm^3^; p < 0.05, all; Fig. 4B,C), which subsequently lead to the compensatory maintenance of flow velocity according to the severity of DS (p = 0.05, Fig. 4D). Microvascular resistance decreased according to the severity of DS (5.4–3.9 mmHg/cm/sec, p = 0.002; Fig. 4E).

In summary, CBF per myocardium (g) decreased with progressive stenosis, although the flow velocity was maintained due to the concomitant reductions in luminal area, volume, and microvascular resistance (Fig. 4F). Interestingly, these curvilinear patterns of physiological parameters were similar to those of invasive physiology studies (Table 3, Supplementary Figure II)^2^.

**Table 3:** Coronary blood flow, flow velocity, and microvascular resistance derived from CCTA.

	N of vessel	CBF (ml/min)	CBF per myocardium (g) (ml/g/min)	Luminal volume (cm^3^)	Mean luminal area (mm^2^)	Flow velocity (cm/sec)	Microvascular resistance (mmHg/cm/sec)	TAG (HU/cm)
All	290	43.7 (28.0–61.2)	0.87 (0.68–1.00)	0.90 (0.62–1.23)	7.38 (5.85– 9.29)	19.0 (14.0–24.6)	4.6 (3.6–6.3)	−16.8 (−24.0–−11.9)
DS = 0%	185	44.2 (27.6–47.6)	0.91 (0.76–1.04)	0.94 (0.61–1.28)	7.80 (6.35–9.70)	18.2 (13.6–23.5)	5.0 (3.9–6.7)	−17.0 (−23.5–−11.8)
DS = 1–49%	34	47.3 (30.3–65.0)	0.84 (0.70–0.98)	0.83 (0.64–1.10)	6.28 (5.36–7.73)	23.6 (17.3–29.6)	3.8 (3.0–5.1)	−14.8 (−22.6–−10.5)
DS = 50–69%	38	37.3 (27.2–64.1)	0.69 (0.59–0.80)	0.80 (0.57–0.96)	6.07 (4.99–7.34)	20.3 (15.0–27.9)	4.0 (3.0–5.5)	−16.0 (−20.1–−13.5)
DS ≥ 70%	33	31.2 (29.3–46.9)	0.64 (0.54–0.76)	0.74 (0.60–1.02)	6.25 (5.70–7.65)	17.4 (14.2–23.5)	3.7 (2.7–4.5)	−24.1 (−27.9–−17.3)
p for trend		0.31	<0.001	0.036	<0.001	0.05	0.002	0.31

p for trend denotes comparison within each column.

**Figure 5:**
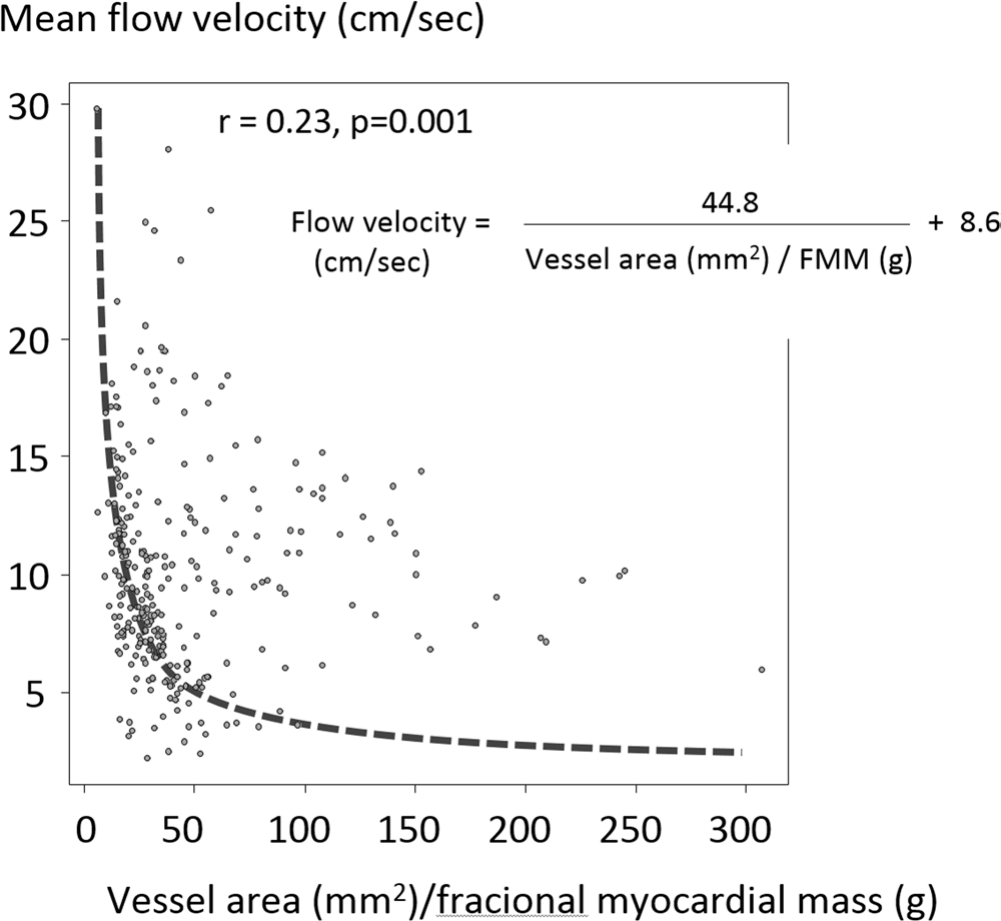
Mean flow velocity versus the ratio of luminal area to fractional myocardial mass. Flow velocity was inversely correlated to the ratio of luminal area to FMM. The coefficient = 44.8 was numerically similar to the coefficient = 46.5, as reported by an invasive Doppler wire study^16^.

Non-invasively acquired parameters were additionally tested for their compliance with the flow continuity principle. Mean flow velocity was inversely related to the ratio of luminal area to regional LV myocardial mass (Equation 2, Fig. 5), which was very similar to the reported by a previous invasive Doppler wire study^16^.

2 $${\rm{Velocity}}(cm/sec)=\frac{44.8}{luminal\,area\,(m{m}^{2})/fractional\,myocardial\,mass\,(g)}+0.86$$

### TAFE versus TAG for detection of DS ≥ 50%

**Figure 6:**
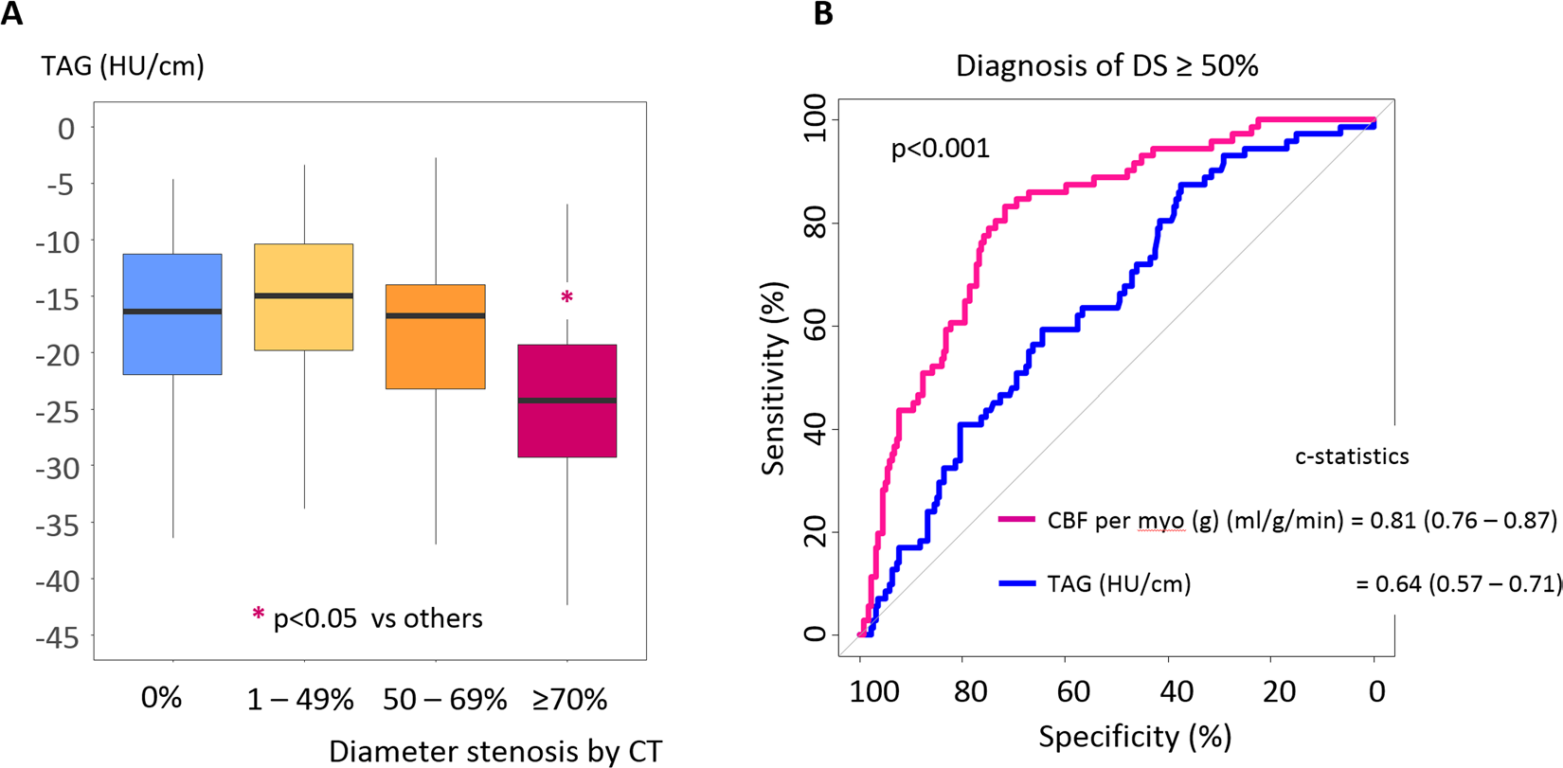
TAFE-derived CBF versus TAG for diagnosis of coronary artery with DS ≥ 50%. (**A**) Only TAG of DS ≥ 70% was significantly lower than the other TAGs (p < 0.05 vs. TAGs of DS = 0%, 1%–49%, or 50%–69%). (**B**) CBF per myocardial mass (g) was better than TAG for the diagnosis of DS ≥ 50%. The optimal cutoff of TAG for DS ≥ 50% was ≤−19.0 HU/cm and showed sensitivity, specificity, positive predictive value, negative predictive value, and accuracy of 59% (47–71), 64% (57–70), 35% (26–44), 83% (76–88), and 63% (57–68).

We tested whether TAFE-derived CBF per myocardium (g) is better than simple intra-arterial iodine profile by TAG for discerning obstructive artery from non-obstructive artery. There was no difference among TAGs of DS = 0%, 1–49%, or 50–69%. Only TAG of DS ≥ 70% was significantly lower than that the other TAGs (p < 0.05, all) (Fig. 6A). The optimal cutoff value of CBF per myocardium (g) (ml/g/min) for DS ≥ 50% was ≤0.82 ml/g/min and the sensitivity, specificity, positive predictive value, negative predictive value, and accuracy were 83% (72–91), 70% (64–76), 48% (39–57), 93% (88–96), and 74% (68–78). The predictive performance of CBF per myocardium (g) for DS ≥ 50% was much higher than that of TAG [c-statistics = 0.81 (0.76–0.87) versus 0.64 (0.57–0.71), p < 0.001] (Fig. 6B).

## Discussion

To the best of our knowledge, this study is the first successful demonstration of comprehensive non-invasive coronary physiology assessment. TAFE-derived CBF was validated in both computational simulation model and human perfusion CT study. In the CCTA study, CBF per myocardium (g) consistently decreased according to a gradual increase in stenosis severity, whereas flow velocity was maintained by the compensatory reduction of microvascular resistance and luminal dimension. These non-invasive study results could replicate the results of previous invasive physiology studies^2,16,17^ and matched well with the counter-intuitively maintained TIMI 3 flow in a wide range of DS (0–90%). Importantly, all these calculations could be performed from readily and rapidly available parameters sourced from the current conventional CT suite without using any stress agents, pre-specified image acquisition conditions, or heavy computational resources. In addition, CBF per myocardium (g) outperformed TAG for the detection of obstructive coronary artery.

### TAFE for non-invasive coronary physiology

CCTA is a snapshot of convection generated by intracoronary time-varying contrast bolus. With the addition of temporal dimension from AIF, TAFE formula can decode the spatial dispersion of contrast concentration along the vessel axis into the temporal dispersion of contrast, which leads to the calculation of CBF^18^. By obtaining further information of regarding vessel dimension and trans-stenotic pressure gradient on the basis of DS, flow velocity and microvascular resistance can be calculated. TAFE can best be compared to an easy-to-use computational flow dynamics condensed in a single formula. A major merit of TAFE is that it does not require any detailed morphological analysis.

Our results provide an insight into why resting flow velocity is maintained in a wide range of stenosis. The decrease of CBF was compensated by offsetting reductions in microvascular resistance and luminal dimension. These findings support the results of recent invasive physiology studies which have demonstrated that both microvessel and epicardial arterial dimension are sensitive to pressure and play a role in coronary autoregulation^2,19^.

### TAFE versus TAG

TAG consistently showed poor correlation with anatomical or physiological stenosis, which has been poorly understood^8–10^. Our findings indicated that TAG reflects the basal CBF rather than the coronary pressure gradient^20^. Our results also explain the discrepancies among TAG and DS or FFR, which otherwise comply with the well-known discordance among the basal CBF, DS, and FFR^2,21^.

### Clinical implications

The major treatment target of coronary artery disease is ischemia-inducing arterial stenosis. In clinical practice, decisions for treatment are often based only on the anatomical angiographic images. However, physiologic study over the past two decades found that mismatch between anatomical and physiological assessment is not uncommon^7,11,17^. Patients without evidence of myocardial ischemia had favorable outcomes without revascularization, and vice versa. That is, physiology guidance is better strategy than anatomy guidance in the treatment of coronary artery disease^22–24^. Hence, invasive physiological assessment with FFR is widely advocated in both American and European clinical practice guidelines for decision of revascularization^25,26^. Despite these well-known clinical benefits, the overall FFR penetration rate is only 6.1% of interventions for intermediate coronary artery stenosis with 40–70% diameter stenosis^27^. This significant underutilization of invasive physiology study suggests that there are still lots of room to expand and to lower the threshold of coronary physiology assessment.

FFR is a simplified pressure-based surrogate for coronary physiology. However myocardium thrives on CBF, not on perfusion pressure^21,28^. Although FFR is the acronym of “fractional flow reserve” and represents a deficit in the maximal flow, FFR is limited as a key physiology surrogate in diffuse atherosclerosis, acute coronary syndrome, diabetes, chronic kidney disease, or microvascular angina, all of which show increased basal CBF or marked variation in arterial luminal size^19,29–33^. In such cases, coronary flow reserve (CFR) and index of microvascular resistance (IMR) have additive or complementary role for prognostic implication^17,34–36^. However, performing comprehensive invasive physiology study that includes the measurements of FFR, CFR, and IMR for all patients is not practical in clinical medicine considering that only a fraction of patients with coronary artery disease receives clinical benefit from catheterization and revascularization^37,38^.

Our methodology enables providing comprehensive physiological data of coronary artery disease, which would expand the utilization and lower the threshold of physiology study and is paramount to good clinical decision-making and improving patient outcome. As additional usage of CCTA-based FFR in clinical practice resulted in less use of invasive procedure and lower healthcare cost compared with conventional practice^38^, CCTA-based comprehensive physiology study may improve the risk stratification of patients, particularly those with diabetes, chronic kidney disease, or diffuse atherosclerosis in which FFR alone may be insufficient for prognostic implication. In addition, non-invasive nature of this methodology enables extraction of physiologic data from preceding CCTA data and large-scaled population-level physiology studies.

### Limitations

Only a single type of CT scanner and a single analyzing workstation was used. As the technical methods and analytical models affect the assessment of positron emission tomography, TAFE coefficient ***k*** may be affected by CT scanners and need calibration for each hardware. TAFE may depend on temporal homogeneity, although comparable intracoronary luminal attenuation results between single-beat and multi-beat scans have been recently demonstrated^39^. The spatial and temporal resolution of CCTA causes partial volume effect and limits the accuracy of TAFE in small vessels, which may be overcome by mathematical correction^18,20,40^. Exclusive assumption of laminar flow may cause overestimation of flow velocity or underestimation of microvascular resistance^15^. Invasive physiology study retrieves pressure and flow data from a single distal point, whereas the region of interest in TAFE is not localized to one point. Finally, non-invasive physiology parameters were not directly compared with invasive measurements.

## Conclusion

TAFE enabled non-invasive quantitative CBF measurement and comprehensive coronary physiology assessment using standard CCTA without any pre-specified condition or complex computational process. TAFE derived CBF was better than TAG for identification of coronary artery with DS ≥ 50%.

## Electronic supplementary material

Supplementary Appendix, Tables, and Figures
