# Supplementary material for: Non-invasive coronary physiology based on computational analysis of intracoronary transluminal attenuation gradient
Source: Sci Rep. 2018 Mar 16;8:4692. doi: 10.1038/s41598-018-23134-7 (PMC5856794; doi:10.1038/s41598-018-23134-7)
Supplement: Supplementary file 1 — Supplementary Appendix, Tables, and Figures [file 41598_2018_23134_MOESM1_ESM.pdf]

Non-invasive coronary physiologic study based on computational analysis of intracoronary transluminal attenuation gradient

- **Vessel-specific coronary blood flow, flow velocity, and microvascular resistance from computational analysis of coronary CT angiography**

Yong Gyun Bae[a], Seung Tae Hwang[a], Han Huan[a], Sung Mok Kim[b], Hyung-Yoon Kim[e], Il Park[c], Joo Myung Lee[c], Young-June Moon[a*], and Jin-Ho Choi[d*]

[a]Computational Fluid Dynamics and Acoustics Laboratory, School of Mechanical Engineering, Korea University, Seoul, Republic of Korea

[b]Department of Radiology, [c]Department of Medicine, [d]Department of Emergency Medicine, Heart Vascular and Stroke Institute, Samsung Medical Center, Sungkyunkwan University School of Medicine, Seoul, Republic of Korea

[e]Department of Medicine, Chonnam National University Medical School, Gwangju, Republic of Korea

Corresponding authors:

Drs. Jin-Ho Choi and Young-June Moon are co-corresponding authors.

Jin-Ho Choi, MD, PhD

Professor, Emergency Medicine, Samsung Medical Center, Sungkyunkwan University

School of Medicine

81 Ilwon-ro, Gangnam-gu, Seoul, Korea

Email: jhchoimd@gmail.com

Phone: 82-2-3410-2053

Young-June Moon, PhD

Professor, Computational Fluid Dynamics and Acoustics Laboratory, School of Mechanical Engineering, Korea University.

Email: yjmoon777@gmail.com

Phone: 82-2-926-3818

Email: jhchoimd@gmail.com

Phone: 82-2-3410-2053

Appendix

Computational simulation model of TAFE

1. Computational methodology

1.1 Simplified coronary artery models

Branching pattern of coronary arteries is schematized as Figure 1. Whole vessel was composed of 1 coronary inlet and 32 outlets with 62 side branches. Fundamental rules of vessel branching pattern include: (1) two branches have same spreading angle (θ1, θ2), and (2) radius of the daughter vessel is determined by power law (Murray's law),

$$r_p{}^{\xi} = r_{\mathrm{d1}}{}^{\xi} + r_{\mathrm{d2}}{}^{\xi}$$

where r_{p}, r_{d1}, r_{d2} represent the radius of parent vessel and two daughter vessels, respectively. We use ξ=3 in simulation. This is a widely suggested ξ value for laminar flow calculation (ξ=7/3 for turbulence flow). Radius of daughter vessel was determined by parent vessel at upstream. Thus geometric characteristics of daughter vessels preserve the characteristics of parent vessel and comply with power law. Branching was recursively done to 6 orders which resulted in 32 branches and total cross-section area ratio between inlet and outlet of 10.079 approximately (Figure 1A-B). Detailed geometric information of artificial coronary arteries from 1st order to 6th order is shown in Table. 1.

Table 1. Dimensional information of coronary arteries

D	L_1	L_2	L_3	L_4	L_5
0.2 cm	12 cm	4 cm	2 cm	1 cm	0.5 cm

We conducted 7 case studies with 1 control group to investigate the impact of diameter stenosis (DS) on the flow characteristics (Table 2). The tendency of flow field variations was investigated by 2-dimensional computational fluid dynamics simulation. The 2D computational domain and detailed grid view is illustrated in Figure 2B. The total number of grid-points is around 1,000,000.

Table 2. Case studies for diameter stenosis

	Reference	Case 1	Case 2	Case 3	Case 4	Case 5	Case 6	Case 7
DS	0%	25%	50%	60%	70%	80%	90%	95%

DS, diameter stenosis (%)

1.2 Fluid model and boundary conditions

The unsteady hydrodynamic flow-field is solved by incompressible Navier-Stokes equations as the following,

$$\frac{\partial \rho}{\partial t} + \nabla \cdot (\rho \vec{U}) = 0$$

$$\frac{\partial\vec{U}}{\partial t} + (\vec{U}\cdot\nabla)\vec{U} - \upsilon\nabla^2\vec{U} = -\frac{1}{\rho}\nabla P$$

where *ρ* represents density, *U* represents velocity vector, *υ* represents kinematic viscosity coefficient, *t* is time, and *P* represents pressure. Blood is modelled as a Newtonian fluid with *μ* = 0.004 *pa* • *s*. We choose the laminar flow as the viscous model since Re=112 is used at the coronary inlet region.

The Reynolds number can be estimated as,

$$Re = \frac{\rho\cdot v_{mean}\cdot d}{\mu}$$

where *ρ* is the density of blood (kg/m^3), *μ* is the dynamic viscosity evaluated at the inlet (*Pa·s*), *v* is the mean velocity at the inlet (m/s), and *d* is the proximal diameter (mm).

Vessel wall is considered as rigid and stationary and no-slip boundary condition (zero velocity) is imposed. Mass flow rate condition is imposed at coronary inlet and constant static pressure outlet condition is prescribed at the 32 branch outlets. Steady calculation method is used for hydrodynamic flow field since the flow rate is constant. But the mass fraction of blood is changed along with arterial input function (AIF). Using AIF which reflects dynamic intravenous contrast distribution and blood flow, the blood flow information could be extracted from contrast distribution snap shots. Demonstrated temporal profile of arterial input function is described in Figure 2C. Therefore we impose

unsteadiness of mass fraction of iodine and it increases until 1.2 % for 15 sec and it lasts 1.2 % for 10 sec which is converted using the ratio of iodine per volume (11.2 mg iodine / ml saline). TAFE measurement moment was t = 25 sec.

2. Results

2.1 Flow field (Iodine distribution)

Vessel-specific CBF was calculated by transluminal arterial flow encoding (TAFE) which consisted of the luminal and axial dimension of vessel and transluminal attenuation gradient (TAG) from single-beat CT data. TAG is generated by the advection of the iodine into the coronary arteries and reflects intracoronary kinematics of iodine. This advection represents the bulk motion of blood and spatial distribution of iodine by the flow characteristics. To investigate the effect of DS, 7 case studies and 1 control group are conducted to how they affect the coronary flow field. TAG measurement position is illustrated in Figure 2D.

TAG is only measured in 1st daughter vessels which represent major CBF dynamics. Total streamline length is normalized as 1 and proximal and distal position compared with total streamline length is 0.1 and 0.65, respectively. The distribution of iodine mass fraction is shown in Figure 2E. This distribution pattern enables prediction of bulk motion of blood flow since contrast distribution is directly related to the velocity or flow rate. There is inverse proportion relationship between diameter stenosis and flow rate. Flow rate is decided by the geometrical resistance because we impose the static pressure condition to outlet. At the stenosis vessel, decrease pattern of contrast distribution is quiet steeper than normal vessel. Gradient of mass fraction of iodine is quantitatively measured along the streamlines in both normal and stenotic vessels. Compared to normal vessel, TAG of stenotic vessel decreased

consistently according to the severity of stenosis (Figure 2F).

2.2 Comparison of flow rate ratio between stenotic and normal vessel

To investigate the effect of stenosis on the TAFE, we compared the flow rate derived from computational fluid dynamics and the flow rate derived from TAFE. As clearly shown in Figure 2G, flow rate ratio between stenotic and normal vessel decreased mildly and gradually until DS<70%, then declined rapidly after DS≥70%. Both flow rate calculated by computational flow dynamics and TAFE agreed well. However when DS≥90%, the flow rate calculated by TAFE did not follow with the flow rate calculated by computational flow dynamics. This mismatch between TAFE and computational flow dynamics would be explained by the nature of TAFE which is the modified transport equation to predict the CBF from TAG. TAFE assumes that the contrast is already passed or just passing the distal vessel at the TAG measurement moment. However, most contrast did not reach to the distal end at the snapshot moment due to severe stenosis and severely decreased flow rate in our simulate model with DS≥90%.

Supplementary Figure I. Computational flow dynamics model

Figure 1. Computational flow dynamics model

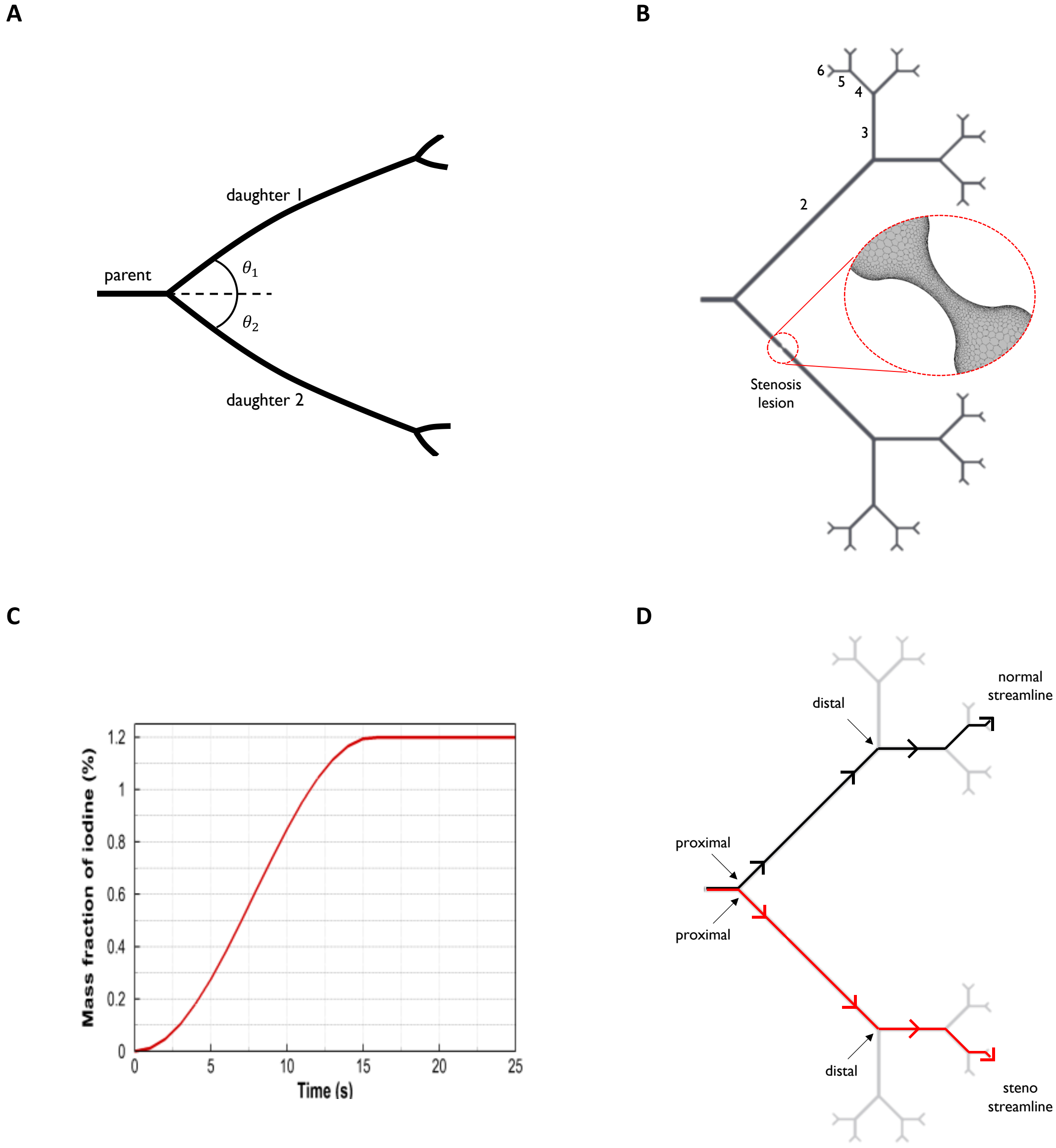

E

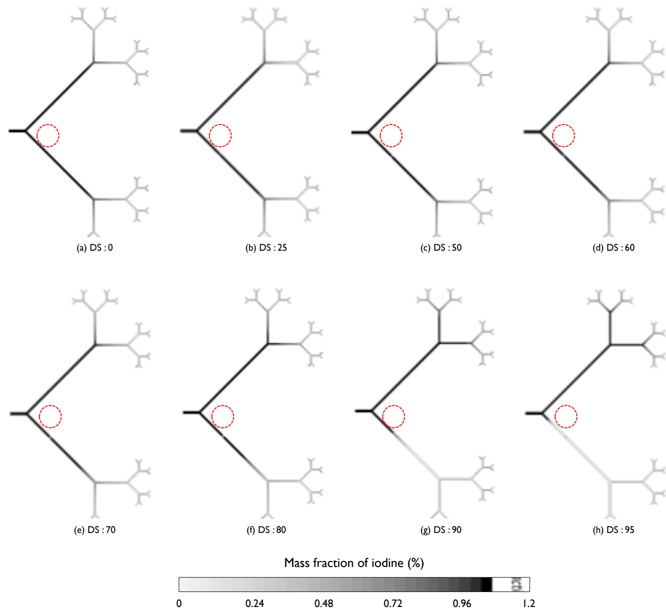
(a) DS : 0
(b) DS : 25
(c) DS : 50
(d) DS : 60
(e) DS : 70
(f) DS : 80
(g) DS : 90
(h) DS : 95
Mass fraction of iodine (%)
0
0.24
0.48
0.72
0.96
1.2

F

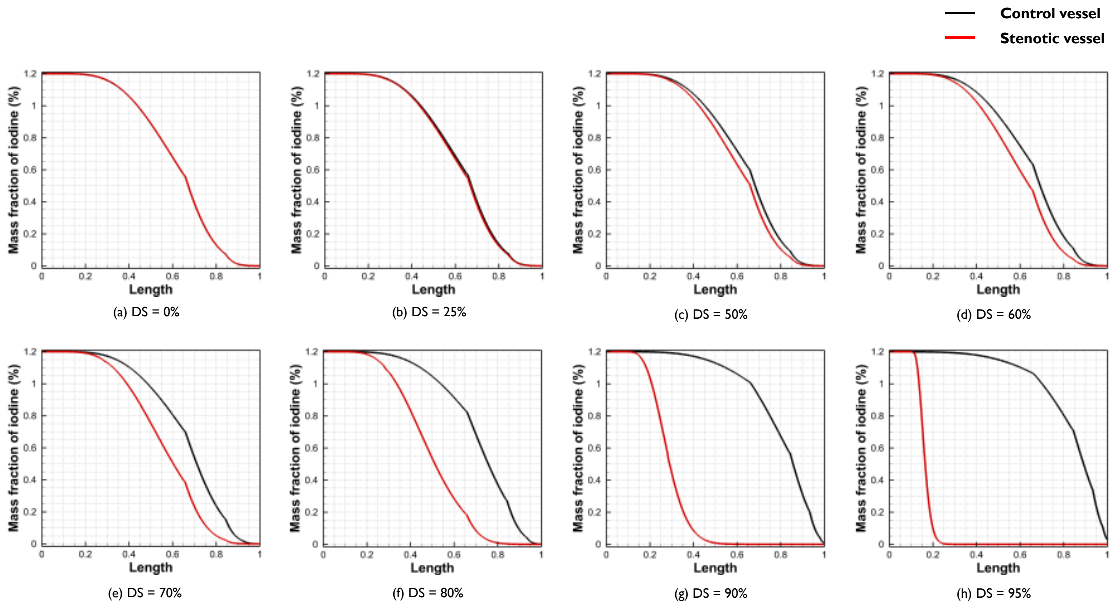
Control vessel
Stenotic vessel
Mass fraction of iodine (%)
Length
(a) DS = 0%
(b) DS = 25%
(c) DS = 50%
(d) DS = 60%
(e) DS = 70%
(f) DS = 80%
(g) DS = 90%
(h) DS = 95%

G

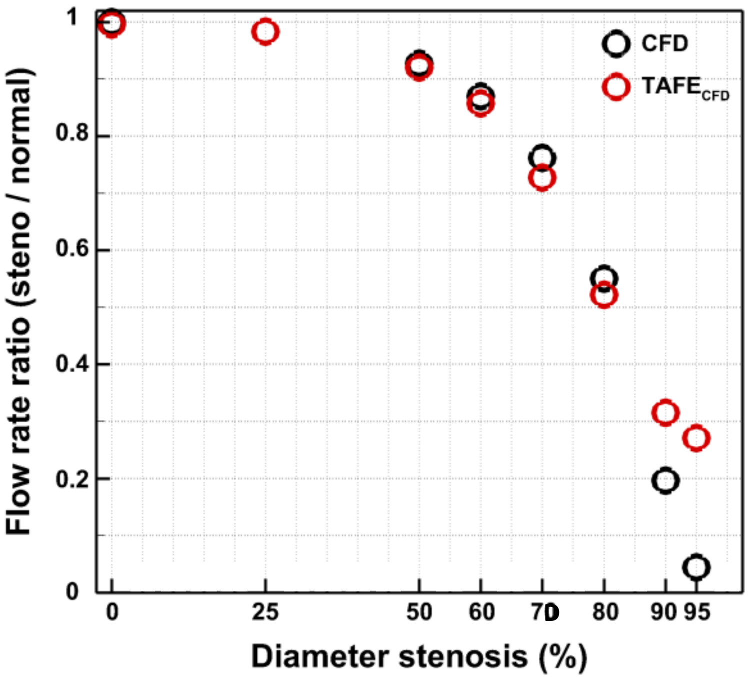

Flow rate ratio (steno / normal)
Diameter stenosis (%)
CFD
TAFE$_{CFD}$
1
0.8
0.6
0.4
0.2
0
0
25
50
60
70
80
90
95

Supplementary Figure II: Comparison of non-invasive coronary physiologic assessment with invasive coronary physiology assessment

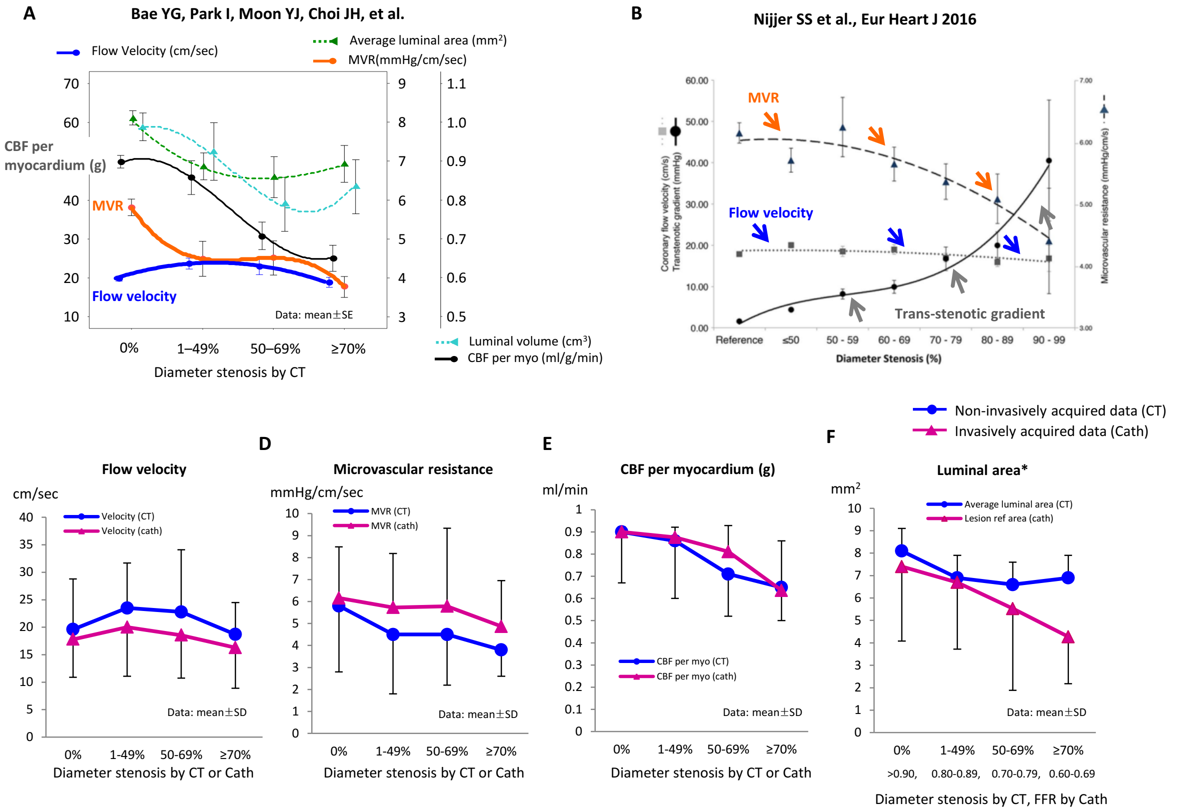

A: CBF per myocardium (g) and microvascular resistance decreased with progressive stenosis, but flow velocity did not change due to concomitant reduction of mean luminal area and volume. Data are mean±SE. Curves were fitted by polynomial quadratic or cubic models.

B: Invasive physiology data from Iberian–Dutch–English (IDEAL) study (Nijjer et al Eur Heart J 2016). With the gradual severity of stenosis, trans-stenotic pressure gradient increased but resting coronary flow velocity is maintained due to compensatory reduction of microvascular resistance.

For panels C-F, statistical comparison was not done because the number of each stenosis category was not shown in the publication of IDEAL study. In case of unknown sample number, overlap of SD error bar does not lead to any statistical conclusion.

C: The pattern of invasive or non-invasively acquired flow velocity. From single-beat CCTA, laminar flow was assumed and flow velocity was determined by 2-fold of TAFE-CBF divided by average luminal area (according to flow dynamics law, maximal flow velocity observed by Doppler wire is exactly 2-fold of mean flow velocity in case of laminar flow). From catheterization data of IDEAL study, flow velocity was directly recorded from Combowire equipped with both Doppler and pressure sensors. Non-linear relation between the severity of stenosis and flow velocity is shown in both data.

D: The pattern of invasive or non-invasively acquired microvascular resistance. From single-beat CCTA, distal mean arterial pressure was calculated by assumed resting whole cycle trans-stenotic pressure gradient based on IDEAL study (1.5, 4.4, 10.8, and 29 mmHg for diameter stenosis (DS)=0%, 1–49%, 50–69%, and ≥70%, respectively) from mean arterial pressure defined by diastolic blood pressure + pulse pressure / 3. Then microvascular resistance was calculated by dividing distal mean arterial pressure to flow velocity. From catheterization data of IDEAL study, microvascular resistance was calculated by dividing distal pressure by flow velocity.

Both non-invasively and invasively acquired microvascular resistance showed decreasing tendency according to the severity of stenosis.

E: The pattern of invasive or non-invasively acquired CBF per myocardium (g). From single-beat CCTA, CBF per myocardium (g) was calculated by TAFE-CBF x %fractional myocardial mass.

From catheterization data of IDEAL study, 0.9 ml/g/min was assumed in vessel with DS=0%. With the assumption of inversed relationship between with flow and total resistance, CBF of IDEAL study was estimated by the product of basal flow and the ratio of trans-stenotic pressure + microvascular pressure of stenotic vessel to trans-stenotic pressure + microvascular pressure of normal vessel.

Both non-invasively and invasively acquired CBF per myocardium (g) showed decreasing tendency according to the severity of stenosis.

F: The pattern of invasive or non-invasively acquired luminal area. * X-axis shows DS for CT, and category of FFR for Cath. Y-axis shows average of proximal and distal luminal area for CT, and lesion reference area calculated by dividing minimal luminal area with (1 – area stenosis) for Cath.

Both non-invasively and invasively acquired CBF per myocardium (g) showed decreasing tendency according to the severity of anatomical or functional stenosis.

Supplementary Figure III: Mean flow velocity versus the ratio of luminal area to fractional myocardial mass, assessed non-invasively and invasively

Bae YG, Park I, Moon YJ, Choi JH, et al.

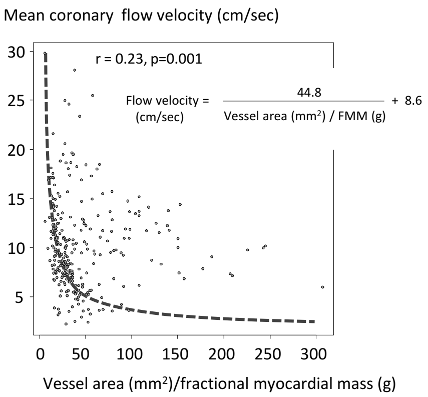

Anderson et al., Circulation 2000

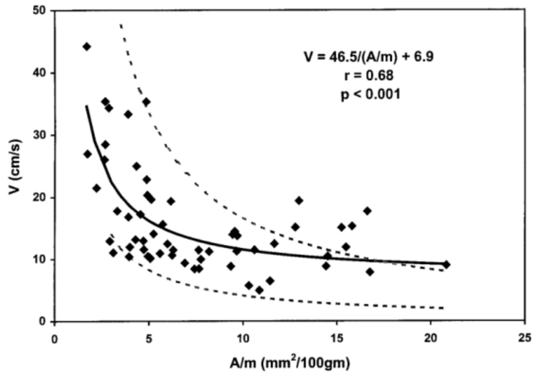

$$\text{Flow velocity (cm/sec)} = \frac{44.8}{\text{Vessel area (mm}^2\text{) / FMM (g)}} + 8.6$$

$$\text{Flow velocity (cm/sec)} = \frac{46.5}{\text{Vessel area (mm}^2\text{) / FMM (g)}} + 6.9$$

Figure 5 is shown here again to be compared with invasive physiology study. Non-invasively acquired parameters were tested whether it comply with the flow continuity principle. Mean coronary flow velocity was inversely related to the ratio of luminal area to regional LV myocardial mass (velocity (cm/sec)= 44.8 / vessel area (mm2) x subtended myocardial mass (g) + 8.6, r=0.23, p<0.001). In a prior invasive physiology study, the correlation coefficient was numerically similar 46.5.
